# Challenges and Complications of Poly(lactic-*co*-glycolic acid)-Based Long-Acting Drug Product Development

**DOI:** 10.3390/pharmaceutics14030614

**Published:** 2022-03-11

**Authors:** Yi Wen Lim, Wen Siang Tan, Kok Lian Ho, Abdul Razak Mariatulqabtiah, Noor Hayaty Abu Kasim, Noorsaadah Abd. Rahman, Tin Wui Wong, Chin Fei Chee

**Affiliations:** 1 Department of Microbiology, Faculty of Biotechnology and Biomolecular Sciences, Universiti Putra Malaysia, Serdang 43400, Malaysia; 1yiwen2@hotmail.my (Y.W.L.); wstan@upm.edu.my (W.S.T.); 2 Laboratory of Vaccines and Biomolecules, Institute of Bioscience, Universiti Putra Malaysia, Serdang 43400, Malaysia; mariatulqabtiah@upm.edu.my; 3 Department of Pathology, Faculty of Medicine and Health Sciences, Universiti Putra Malaysia, Serdang 43400, Malaysia; klho@upm.edu.my; 4 Department of Cell and Molecular Biology, Faculty of Biotechnology and Biomolecular Sciences, Universiti Putra Malaysia, Serdang 43400, Malaysia; 5 Faculty of Dentistry, Universiti Kebangsaan Malaysia, Jalan Raja Muda Abdul Aziz, Kuala Lumpur 50300, Malaysia; nhayaty@ukm.edu.my; 6 Institute for Advanced Studies, Universiti Malaya, Kuala Lumpur 50603, Malaysia; noorsaadah@um.edu.my; 7 Non-Destructive Biomedical and Pharmaceutical Research Centre, Smart Manufacturing Research Institute, Universiti Teknologi MARA, Puncak Alam 42300, Malaysia; 8 Nanotechnology and Catalysis Research Centre, Universiti Malaya, Kuala Lumpur 50603, Malaysia

**Keywords:** poly(lactide-*co*-glycolide), drug delivery, sustained release, PLGA microspheres, long acting injectable/implantable, complex generic drug products

## Abstract

Poly(lactic-*co*-glycolic acid) (PLGA) is one of the preferred polymeric inactive ingredients for long-acting parenteral drug products that are constituted of complex formulations. Despite over 30 years of use, there are still many challenges faced by researchers in formulation-related aspects pertaining to drug loading and release. Until now, PLGA-based complex generic drug products have not been successfully developed. The complexity in developing these generic drug products is not just due to their complex formulation, but also to the manufacturing process of the listed reference drugs that involve PLGA. The composition and product attributes of commercial PLGA formulations vary with the drugs and their intended applications. The lack of standard compendial methods for in vitro release studies hinders generic pharmaceutical companies in their efforts to develop PLGA-based complex generic drug products. In this review, we discuss the challenges faced in developing PLGA-based long-acting injectable/implantable (LAI) drug products; hurdles that are associated with drug loading and release that are dictated by the physicochemical properties of PLGA and product manufacturing processes. Approaches to overcome these challenges and hurdles are highlighted specifically with respect to drug encapsulation and release.

## 1. Introduction

PLGA is considered as one of the best inactive ingredients for drug formulation owing to its biodegradability and tunable properties [1]. It has been approved by the U.S. Food and Drug Administration (FDA) and the European Medicines Agency (EMA) for drug delivery and many other biomedical applications. PLGA-based LAI drug products allow extended release over long periods of time and require low dosing frequency [2], thereby increasing patient compliance [3,4,5]. To date, 25 PLGA-based long-acting drug products, all in the injectable or implantable forms, have been approved by the FDA (Table 1) [6]. However, these PLGA-based LAI drug products are relatively expensive for most patients [2]. Although many of these PLGA-based LAI drug products having become off-patent or non-exclusive, no generic version of these drug products is available in the market [7,8]. This situation suggests that it is difficult to develop generic PLGA-based long-acting drug products that involve complex formulations and manufacturing processes. It is difficult for the generic pharmaceutical industry to replicate existing PLGA-based LAI drug products.

## 2. Complexity in Developing Generic PLGA-Based LAI Drug Products

In order for a generic PLGA-based LAI drug product to obtain approval in the Abbreviated New Drug Application (ANDA), the generic candidate is required to achieve qualitative and quantitative (Q1 and Q2) sameness as the reference listed drug (RLD) [16]. In other words, generic drug manufacturers have to prove that their generic PLGA-based long-acting drug products are pharmaceutically, therapeutically, and biologically equivalent to the RLD. The generic drug products should have the same API, dosage form, strength, administration route, absorption rate of the API, safety profile, and efficacy as the RLD [2]. Failing to do so would likely end up in suspension of marketing authorisation, such as the cases of Novosis Goserelin, Goserelin Cell Pharm, and Novimp [17].

One of the limiting factors for creating biosimilar PLGA-based LAI drug products is the complexity of the manufacturing process. Minor modifications in the manufacturing process, involving quality assurance/quality control (QA/QC) systems, can create a major impact in terms of efficacy, bioactivity, stability, and safety of the product [18]. For example, Nutropin Depot^®^, a treatment for growth hormone deficiency in children, has been withdrawn from the market due to manufacturing issues [19]. There is a need for a standard compendial in vitro method for measuring the drug release profiles [20]. Equivalent Q1/Q2 as the RLD does not mean that generic PLGA-based LAI drug products have the same in vitro or in vivo pharmacokinetic profiles as RLD, owing to the sameness potentially being derived from the test methods of differing protocols [16]. The establishment of a validated universal testing protocol, as well as a systematic manufacturing process, is imperative for comparison between the generic PLGA-based LAI and the RLD.

Generally, the manufacturers of generic PLGA-based LAI drug products need about 2000–5000 doses of the RLD to run tests in order to prove their generic drug products are the same as RLD [21]. However, the supply of RLD has been limited. Some drug companies might use regulatory restrictions or commercial tactics to block the supply of RLD to generic drug companies [21]. One of the common anti-generic strategies involves exploitation of the citizen petitions to section 505(q) of the Federal Food, Drug, and Cosmetic Act to delay approval of a pending ANDA [21]. The drug companies might use their commercial contracts or agreements with the distributors to restrict the drug supply to generic drug companies [21]. Such limited distribution may have been imposed by the drug companies as part of a drug safety program (i.e., Risk Evaluation and Mitigation Strategy) implemented by the FDA [21].

To assist the generic pharmaceutical companies in their ANDA application, the FDA publishes product-specific guidance (PSG) describing the Agency’s expectations on the development of generic drug products that are therapeutically equivalent to a specific RLD [22]. The PSG contain information about the recommended bioequivalence studies, dissolution test methods, sampling times, expectations and evidence that are required to support the ANDA approval. However, not all PSG are available for the 25 approved PLGA-based LAI drug products. In this connection, a regulatory science research programme under the Generic Drug User Fee Amendments (GDUFA) was established to support PLGA-based complex generic drug product development [12]. Several grants and contracts have been warranted for the following areas: (a) in vitro–in vivo correlations (IVIVC), (b) in vitro release testing methods, (c) characterisation of PLGA and polylactic acid (PLA), (d) modelling and simulation of PLGA/PLA-based drug products, (e) protein–PLGA interactions, (f) separation of PLGA polymeric mixture, and (g) impact of PLGA properties on product performance [23]. While outcomes from these studies are yet to be fully used as a guidance for industry, the generic drug companies are encouraged to discuss with FDA for any new method/solution pertaining to these areas of focus. As the medicines regulatory authority, EMA has set up a recommended framework to follow, which includes the comparative quality, clinical and non-clinical studies [24]. Overall, the FDA and the EMA are consistently establishing the proper standards and guidelines for the development of PLGA-based complex generic drug products.

Nevertheless, even if generic drug developers have overcome the above-mentioned challenges, the anticipated return would not be good enough to compensate for the amount of time and resources spent on the development of PLGA-based complex generic drug products. In this scenario, generic drug developers would rather submit their newly developed PLGA-based long-acting drug products as a new drug application (NDA) with a new indication, dosage form or strength. The return of filing an NDA is envisaged to be higher than that of the generic version. To assist in generic product development that provides more affordable healthcare, this review aims to highlight challenges in association with materials, formulations, processing and testing attributes and provide an insight to possible solutions and strategies.

## 3. Lack of a Standard Compendial Method for In Vitro Release Studies

PLGA- and PLA-based polymers have been exploited for the delivery of a broad class of APIs, including small-molecule drugs, proteins, peptides, and nucleic acids, covering a wide range of treatments listing from cancers, endocrine disorders, psychiatric illnesses, to periodontal diseases [25]. Despite over 30 years of development along with 25 approved formulations, a standard compendial method with which a new PLGA formulation can be compared has yet to be established [20,26]. Each of the PLGA-based LAI drug products has its own unique features, and is specifically tailored to fit certain desired characteristics and demands, such as suitable particle size, high drug loading capacity and controlled release profile [27]. Among the common drug delivery systems used for PLGA-based long-acting drug products, such as microparticles, nanoparticles, micelles, drug conjugates, and LAI, only microparticles and LAI have been approved by the FDA [12]. Each PLGA-based long-acting drug product has its individual required drug release/action profiles as a function of route of administration, dosage form design (i.e., size, shape, surface charge, drug interaction, and inclusion of targeting moiety), drug load, and drug properties (i.e., solubility, stability, potency, site of action, and clearance rate). Each of these parameters significantly impacts the overall performance of PLGA-based long-acting drug products and their distribution in the body. Without fully understanding the material (drug and polymer) and product properties, it will be difficult to achieve the desired therapeutic outcomes.

Due to the abovementioned complexities, the development of in vitro release testing and the establishment of IVIVC have been challenging [26]. There is a need for a compendial in vitro release method to analyse PLGA-based complex generic drug products [18]. An ideal in vitro release testing method should have good reproducibility and the ability to discriminate between different PLGA formulations as well as different production batches of a formulation, should the latter be met with a variation in product quality. The method should allow inter-laboratory comparisons [18].

**Figure 1:**
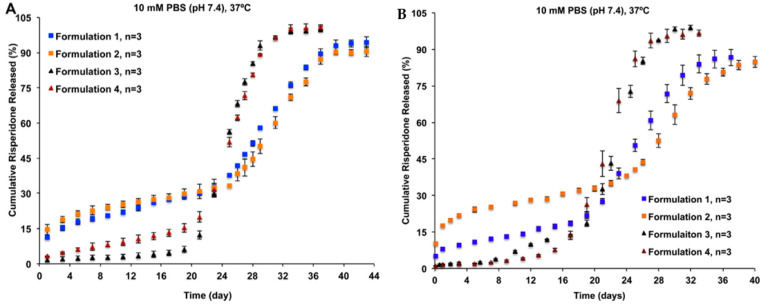
(**A**) The sample-and-separate method, and (**B**) the continuous flow method used in discrimination of the in vitro release profiles of risperidone from four PLGA microsphere formulations with equivalent compositions but different manufacturing processes. In comparison to the sample-and-separate method, the continuous flow method can better differentiate the release of risperidone ascribing to the porosity of microspheres (i.e., Formulations 3 and 4 vs. Formulations 1 and 2), as well as their particle sizes (i.e., Formulations 1 vs. 2). (Reprinted from *Journal of Controlled Release*, 218, Jie Shen, Stephanie Choi, Wen Qu, Yan Wang and Diane J. Burgess, In vitro-in vivo correlation of parenteral risperidone polymeric microspheres, 2–12, Copyright (2015), with permission from Elsevier [18]).

Conventional in vitro release methods, such as dialysis, sample-and-separate, and continuous flow methods, have been evaluated for their suitability for analysing PLGA-based LAI drug products [18]. In a release study of four risperidone-loaded PLGA microsphere formulations of different particle porosity and size, the continuous flow method showed better differentiation against all formulations than the sample-and-separate method (Figure 1) [18]. The continuous flow method was able to differentiate the release of risperidone ascribing to the porosity of microspheres (i.e., Formulations 3 and 4 vs. Formulations 1 and 2), as well as their particle sizes (i.e., Formulations 1 vs. 2).

An accelerated in vitro release study at 45 °C using the continuous flow method was developed for PLGA microspheres with equivalent compositions under different manufacturing processes [28]. The continuous flow method can differentiate three risperidone microsphere formulations with different porosity attributes from their accelerated in vitro release profiles. The accelerated in vitro release study was developed to suit labile API such as naltrexone, which degrades by more than 30% over 30 days in phosphate buffer saline solution (pH 7.4) [29]. It was found that higher temperature allows faster polymer erosion and drug diffusion, thereby accelerating drug release within a shorter period of time. However, an elevated temperature study resulted in the degradation of naltrexone. To overcome this problem, a small amount of sodium ascorbate can be added as an antioxidant to suppress the degradation of naltrexone.

Elevated temperature accelerated release studies may not be suitable for poorly soluble drugs, such as triamcinolone and dexamethasone. This is because the release kinetics of poorly soluble drugs is slower at elevated temperatures [29,30,31]. It has been postulated that PLGA plasticisation occurs at elevated temperatures, resulting in the closure of the microspheres’ internal channels and surface pores [32]. The elevated temperature also facilitates drug recrystallisation within the microspheres. The summation of these effects leads to reduced drug release.

For each given drug, the PLGA-based long-acting product may have its own release and IVIVC profiles. The established IVIVC may be used as a surrogate for bioequivalence (BE) studies, thus reducing the time and resources required for generic drug product development [20]. Among the three primary IVIVC categories, i.e., A, B, and C, level A is the most common type of IVIVC because it contains a point-to-point correlation between the in vitro dissolution rate and the in vivo input rate. Therefore, the FDA recommends establishing level A IVIVC using at least two formulations with different release kinetics [33].

Level A IVIVCs have been successfully developed in rabbits for PLGA microspheres that are equivalent in formulation composition containing small-molecule drugs (e.g., naltrexone) [20], as well as peptides (e.g., leuprolide) [34]. The developed IVIVCs can not only detect in vitro performance changes (i.e., release characteristics) resulting from manufacturing process differences, but can also predict the in vivo performances of the microspheres. When the in vivo pharmacokinetic profiles (fraction absorbed/released) of Vivitrol^®^ were predicted from its in vitro release profile using three different developed IVIVCs, all three predicted in vivo pharmacokinetic profiles were similar to the experimental in vivo pharmacokinetic profile obtained in rabbits [20]. However, the developed IVIVCs were specifically tailored for naltrexone-loaded microspheres (i.e., Vivitrol^®^), and were not suitable for other small-molecule APIs with different release profiles.

It was recently reported that IVIVCs developed using PLGA microsphere formulations with consistent in vitro burst release characteristics demonstrate better predictability with respect to their in vivo pharmacokinetics profile [35]. Seven compositionally equivalent risperidone and five compositionally equivalent leuprolide acetate formulations were prepared using different solvent systems and mixing methods. The resulting PLGA microspheres exhibited varying burst release profiles due to differences in particle size and porosity. The formulations were grouped into low and high burst release, respectively, for the development of IVIVCs. As a result, the IVIVCs developed using low-burst-release formulations showed good predictability for formulations with low burst release and vice versa. The IVIVCs developed using low-burst-release formulations are not suitable for the prediction of formulations with high burst release.

## 4. Constraints Related to the Physicochemical Properties of PLGA

The physicochemical properties of PLGA affect drug release and their influences have been well studied in vitro [36,37,38]. Examples of this include PLGA composition (ratio of lactide to glycolide), molecular weight (MW) and weight distribution, polymer architecture (e.g., linear or star-shaped), polymer end-cap, crystallinity, glass transition temperature (Tg), porosity, particle size, particle size distribution, surface morphology, drug content, hydrophilicity, and hydration rate [18]. To facilitate the reading, a summary of the constraints related to the physicochemical properties of PLGA during its formulation in the development of LAI drug products is listed in Table 2.

Studies have shown that PLGA with a lactide-to-glycolide ratio of 50:50 has the fastest biodegradation rate (50–60 days) [1]. Due to the hydrophilic nature of glycolic acid, PLGA composition with a higher proportion of glycolic acid has a higher hydration rate [39]. On the other hand, PLGA composition with a lower proportion of glycolic acid gives rise to a slower drug release rate. PLGA characterised by a smaller particle size (200 nm) degrades faster in vivo compared to in vitro [38]. In smaller PLGA particles, the water uptake is higher because of a shorter diffusion diameter [40]. In larger PLGA particles, the degraded oligomers have a longer path to diffuse out from the internal of the particles to the surface, during which autocatalysis of PLGA can affect pH changes and drug instability [41].

The MW of PLGA is considered to be one of the significant factors affecting drug release. It can influence drug release rate and its pharmacokinetic profiles [42,43]. PLGA with a higher MW generally tends to have a slower degradation rate and drug release kinetics, as it needs more time to hydrolyse into soluble oligomers [45]. Low-MW PLGA (16 kDa) has nonetheless been demonstrated to show a lower burst release than that of high-MW PLGA (60 kDa), as uneven drug distribution takes place in the latter [46]. High-MW PLGA may translate to the formation of a larger matrix. Large PLGA microspheres increase propensity for drug release due to the higher drug loading and drug-induced porous structure, which confers a higher drug diffusion rate [47,48]. Octreotide acetate has been used as a model drug for studying the drug release behaviour of microspheres prepared from different blends of high- and low-MW PLGA [49]. When low-MW PLGA (5 kDa) is blended with high-MW PLGA (51 kDa) at a weight ratio of 3:7, the resulting mixed PLGA microspheres are characterised by a lower burst release compared to that of commercial Sandostatin Lar^®^. A blend of low-MW PLGA reduces the rate of polymer precipitation during microsphere solidification, which enables the formation of a dense polymer matrix. As a result, the mixed PLGA microspheres experience a more effective drug encapsulation and a lower rate of drug diffusion.

**Figure 2:**
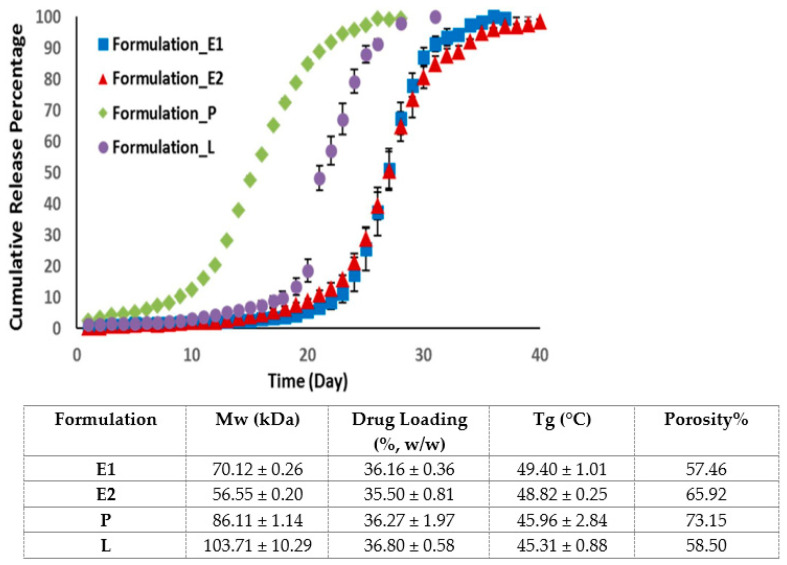
Cumulative release of risperidone from four microsphere formulations with similar drug loading but different porosities and glass transition temperatures. Formulation P, with a high porosity % but a low Tg, displayed the fastest drug release. (Adapted from *International Journal of Pharmaceutics*, 582, Moe Kohno, Janki V. Andhariya, Bo Wan, Quanying Bao, Sam Rothstein, Michael Hezel, Yan Wang and Diane J. Burgess, The effect of PLGA molecular weight differences on risperidone release from microspheres, 119339, Copyright (2020), with permission from Elsevier [43]).

Contrary to previous studies, an in vitro release study of four PLGA microsphere formulations with similar drug loading showed that the drug release is independent of the MW of PLGA [43]. Indeed, the drug release is dependent on the glass transition temperature and the porosity of the microspheres (Figure 2) [43]. Formulations with a higher porosity allow more water to penetrate into PLGA microspheres, which accelerates polymer degradation and drug release. Formulation with a lower Tg is characterised by a higher PLGA chain flexibility, which enhances the water accessibility and drug release of the microspheres.

**Figure 3:**
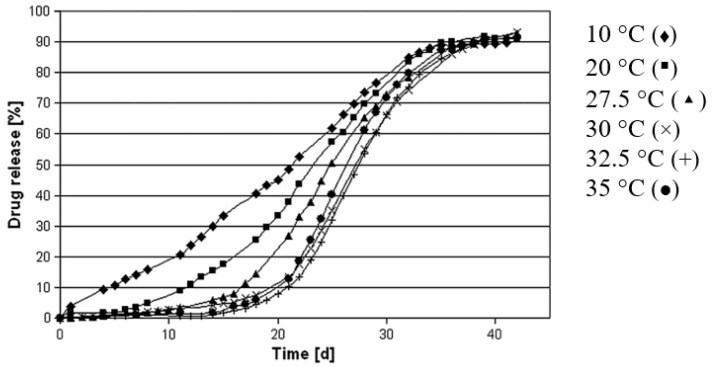
Drug release profiles of PLGA microspheres prepared under varying temperatures using emulsification solvent extraction/evaporation technique. High processing temperature results in a longer lag phase and a more pronounced sigmoidal drug release profile. (Reprinted from *European Journal of Pharmaceutics and Biopharmaceutics*, 81, Kerstin Vay, Wolfgang Frieß and Stefan Scheler, A detailed view of microparticle formation by in-process monitoring of the glass transition temperature, 399–408, Copyright (2012), with permission from Elsevier [42]).

The glass transition temperature of PLGA dictates the particulate microstructure and drug release. The processing temperature of PLGA polymer matrix higher than its Tg temperature produces PLGA microspheres with a dense matrix and a smooth surface, whereas the processing temperature lower than its Tg temperature produces porous microspheres [42]. Temperatures higher than Tg of PLGA may result in an increase in chain plasticity, allowing polymer rearrangement into a dense matrix. As a result, the release profiles of PLGA microspheres made at higher temperatures exhibit a lag phase followed by a burst release profile (Figure 3).

The degradation rate of PLGA may impact the release of drug from PLGA microspheres, and is likely affected by the surrounding pH, temperature, and additive [1,50]. Acidic pH conditions (pH 2.4) have been found to exert little effect on the burst release behaviour of PLGA microspheres [51]. The use of pH-modifying excipients, such as magnesium hydroxide or acetate, has an insignificant impact on PLGA degradation [52]. The introduction of triethyl citrate was found to accelerate the in vitro release of a PLGA formulation containing a hydrophobic drug, triamcinolone acetonide, due to increased mobility and higher diffusion of the polymer in release media [50]. Autocatalysis plays an important role in PLGA degradation [53,54]. During degradation, hydrolysis of the PLGA produces lactic and glycolic acids by-products. The accumulation of these acidic by-products within PLGA microspheres further catalyses the hydrolysis of PLGA, resulting in the formation of a network of pores and channels for drug release [55], detectable by using low-temperature scanning electron microscopy (cryo-SEM) [56]. The release profile of PLGA formulations is also governed by the drug–polymer interaction, as in the case of Sandostatin LAR^®^. In the presence of the acid terminal PLGA linear chain, the interactions between octreotide and PLGA become so significant that they result in bond breaking and formation, i.e., acylation of octreotide [8]. The interaction between octreotide and linear PLGA chains prevents the release of peptides during the initial release phase [8].

PLGA microspheres used for osteoarthritis knee pain may be subjected to degradation due to the static pressure of the joint cavity and shearing force. A static pressure of 4.0 MPa can accelerate polymer degradation and drug release [57]. PLGA:drug weight ratio is also an important factor influencing the release of PLGA particulates. PLGA microspheres with a low drug loading exhibit a distinct tri-phasic release profile, while those with high drug loading mostly display mono- or bi-phasic release kinetics [10,58]. With reference to protein drugs, which are macromolecular therapeutics, their encapsulation by PLGA alone is characterised by the surface deposition and initial burst release of protein drugs. PLGA by itself has insufficient viscous forces to retain the protein molecules in the core of the microspheres. The protein drug encapsulation may be improved by incorporating hyaluronic acid (HA) into PLGA microspheres [59]. The addition of HA increases the viscosity of the PLGA matrix, resulting in a decrease in the diffusion of protein molecules to the surface of PLGA microspheres during processing, thereby reducing burst release tendency.

The type of polymer (i.e., star or linear branched chain) may affect the release characteristics of drugs. Conventional analytical methods, such as gel permeation chromatography and nuclear magnetic resonance spectrometry, are capable of characterising MW, lactide:glycolide (L:G) ratio, and end-group of linear chain PLGA formulations, but they may not adequately characterise star-branched PLGA formulations. Recognising this limitation, an analytical method based on gel permeation chromatography with multiple detectors (i.e., refractive index detector, viscometer, and light scattering detector) was developed to characterise branched PLGA polymer, particularly the number of branches per molecule [44]. The method can not only differentiate the glucose-star PLGA polymer from Sandostatin LAR^®^, but also other branched PLGA from different manufacturers. This makes it possible to relate the drug release property to the type of polymer.

## 5. Complex Drug Release Mechanism

**Figure 4:**
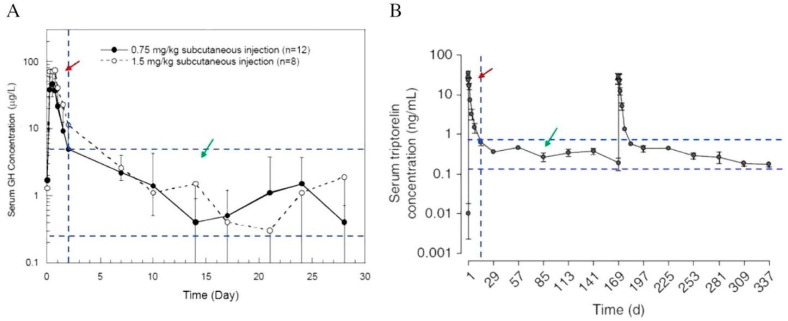
Pharmacokinetic profiles of Nutropin Depot (**A**) and Trelstar (**B**). The red arrows indicate the initial burst release region, and the green arrows indicate the therapeutically effective region. In both profiles, the serum drug concentrations in the initial burst release region are much greater than that of the therapeutically effective region (Reprinted from *Journal of Controlled Release*, 219, Yeon Hee Yun, Byung Kook Lee, Kinam Park, Controlled Drug Delivery: Historical perspective for the next generation, 2–7, Copyright (2015), with permission from Elsevier [60]).

It has been reported that most of the early developed PLGA-based injectable depot formulations have initial burst release characters, resulting in high drug concentration in the body a few days after the injection (Figure 4) [18,60]. The undesired in vivo burst release from PLGA microspheres could lead to the development of severe adverse effects. On this note, understanding the mechanism of drug release is of utmost importance [61]. However, the release profiles of PLGA-based LAI drug products are highly complex and are greatly affected by PLGA characteristics and their interaction with drugs, excipients, the surrounding medium, pH, and temperature. In most cases, the drug release profiles can be identified by comparing the outcomes of in vitro study and in vivo investigation, as inferred from pharmacokinetic study, as well as between different animal models (rat, rabbit, human) of the latter. The factors affecting the drug release and matrix degradation of PLGA microspheres in vitro and in vivo have been discussed previously [45,62,63]. Prior to the development of a reliable in vitro release method, it is important to understand the mechanism of “release” from a PLGA-based LAI drug product in vivo. This information is available either on the basis of reported data, or through investigations performed by the respective generic drug makers.

**Figure 5:**
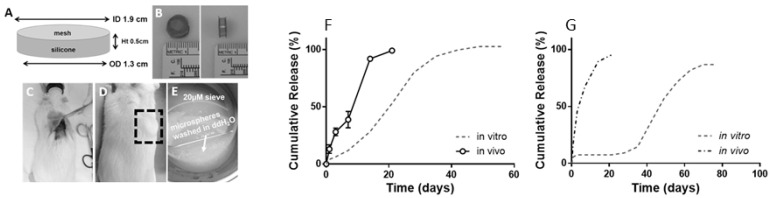
(**A**) Dimensions of a cage implant. (**B**) Top and side view of the silicone rubber/stainless cage implant. (**C**,**D**) Cage that is subcutaneously implanted in a rat (**E**) Retrieved cage prior to analysis (Reprinted from *Biomaterials*, 109, Amy C. Doty, Keiji Hirota, Karl F. Olsen, Naoya Sakamoto, Rose Ackermann, Meihua R. Feng, Yan Wang, Stephanie Choi, Wen Qu, Anna Schwendeman, Steven P. Schwendeman, Validation of a cage implant system for assessing in vivo performance of long-acting release microspheres, 88–96. Copyright (2016) with permission from Elsevier [61]); (**F**,**G**) Release of triamcinolone acetonide and leuprolide, respectively in vivo and in vitro in pH 7.4. A faster-than-expected drug release was observed from both PLGA formulations in vivo (Reprinted from *Journal of Controlled Release*, 256, Amy C. Doty, David G. Weinstein, Keiji Hirota, Karl F. Olsen, Rose Ackermann, Yan Wang, Stephanie Choi, Steven P. Schwendeman, Mechanisms of in vivo release of triamcinolone acetonide from PLGA microspheres, 19–25, Copyright (2017), with permission from Elsevier [36]).

One of the challenges in probing the release mechanism is to retrieve the PLGA formulation after in vivo administration. A group of researchers used cage implants for degradation and drug release studies from PLGA microspheres. The cage is designed in such a way that the introduction of microspheres is simple, and the retrieval of microspheres is possible when necessary. A silicone rubber/stainless steel cage implant system was developed for the establishment of IVIVCs of two different triamcinolone acetonide-loaded PLGA formulations in rats (Figure 5) [61]. The cage was implanted subcutaneously, recovered, and analysed for release kinetics and mass loss. Subsequently, release of the API was compared to the in vitro data. The results showed that faster-than-expected drug release could be observed from the PLGA microspheres in vivo (Figure 5). The rate of PLGA degradation increased in vivo. However, inflammatory responses were observed 2 weeks after the cage implantation in vivo. The inflammatory response may further promote PLGA degradation, thereby increasing drug release.

**Figure 6:**
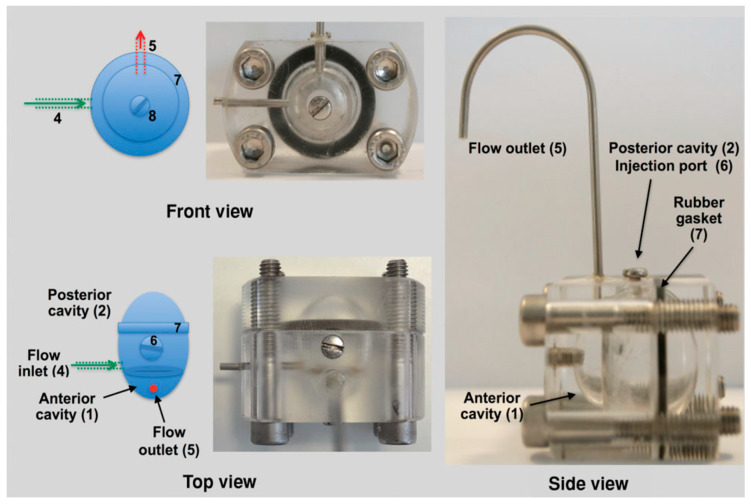
A two-compartment in vitro model of the eye (known as PK-Eye) used in study of the release profile of dexamethasone loaded PLGA microparticles. (Reprinted from *Journal of Pharmaceutical Sciences*, 104, Sahar Awwad, Alastair Lockwood, Steve Brocchini, Peng T. Khaw, The PK-Eye: A novel in vitro ocular flow model for use in preclinical drug development, 3330–3342, Copyright (2015), with permission from Elsevier [67]).

Dialysis membrane and continuous flow methods have traditionally been used for in vitro release studies of ocular formulations [64,65]. The main limitation of these methods is that they do not resemble the human ocular aqueous flow [66]. To overcome this limitation, a two-compartment in vitro model of the eye (known as PK-Eye) (Figure 6) was developed to determine the release kinetics of small-molecule drug as well as protein drug-loaded PLGA microparticles [64,67]. The clearance times obtained from the in vitro model study were then used together with the published human ocular pharmacokinetics data to establish an IVIVC for intraocular clearance times of the PLGA microparticles. The results indicated that the model could be used to develop an IVIVC for ocular formulations.

## 6. Differences in the Manufacturing Process

Various technologies have been applied in the fabrication of drug-loaded PLGA particulates. Conventional emulsification techniques such as water-in-oil (W/O), oil-in-water (O/W), and water-in-oil-in-water (W/O/W) have been widely used owing to their inexpensiveness and the ease of controlling process parameters [68]. Switching from one of these emulsion-based techniques to another significantly affects the properties of the obtained PLGA particles in terms of drug loading, drug encapsulation efficiency, and drug release behaviour. For instance, triptorelin acetate-loaded PLGA microspheres have been developed using the liquid-in-oil-in-oil (L/O/O) emulsification method. They are characterised by higher drug loading and encapsulation efficiency with reduced initial burst release compared to that of prepared by solid-in-oil-in-oil (S/O/O) method [69]. This is attributed to the better solubility of triptorelin acetate in acetic acid. The globule phase of emulsion, which allows it to be efficiently encapsulated in PLGA using the L/O/O method, exhibits a very low initial burst release tendency followed by a sustained release phase of drug [69].

Studies have shown that heterogeneous emulsification generates PLGA microspheres with various sizes [70]. PLGA particulates produced by emulsification techniques often show batch-to-batch variation due to the lack of control in the mixing process [71]. The non-uniformity of the particle size causes inconsistent drug release, which affects the overall efficiency of drug delivery [72]. PLGA particulates synthesised on a batch-to-batch basis using emulsion solvent evaporation and electrospraying methods have constantly suffered from low production yield and scaled up reproducibility [73,74]. The challenges came from the alteration of production conditions and inadequate control of mixing, heat and/or mass transport during the preparation process [75]. Additionally, it is difficult to generate homogeneous PLGA particulates using the emulsion solvent evaporation technique. The processing parameters of this technique, such as shearing rate, and stirring rate require stringent control; otherwise, they will produce PLGA particles with varying sizes [76]. Progesterone-loaded PLGA microparticles prepared by emulsion solvent evaporation and electrospraying methods are different in size, with the electrospraying method generating smaller particle sizes under a high voltage electrical field [27,77].

**Figure 7:**
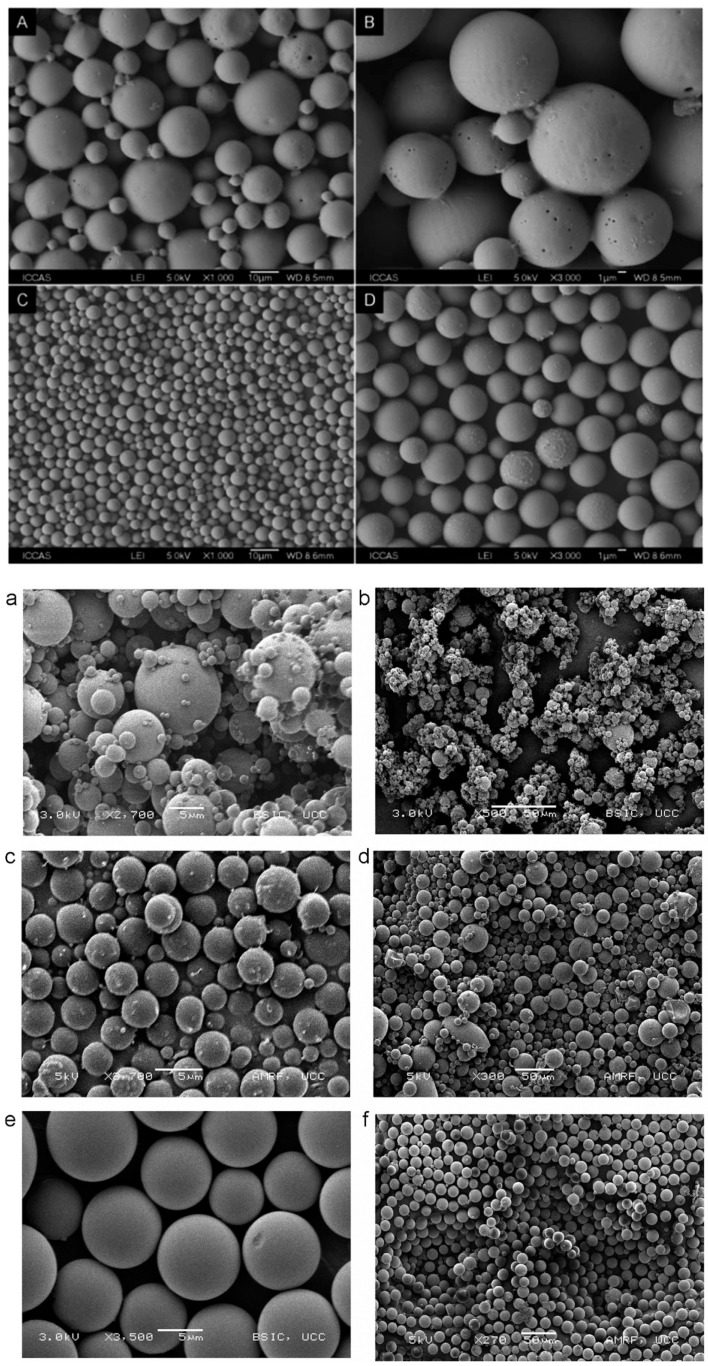
Scanning electron microscopic images of PLGA microparticles prepared using various processing techniques: (**A**,**B**) traditional W/O/W technique, (**C**,**D**) membrane emulsion method (Reproduced from *Polymer Chemistry* (2014), 5, Baoxia Liu, Xiao Zhou, Fei Yang, Hong Shen, Shenguo Wang, Bo Zhang, Guang Zhi, Decheng Wu, Fabrication of uniform sized polylactone microcapsules by premix membrane emulsification for ultrasound imaging, 1693–1701, with permission from the Royal Society of Chemistry [78]); (**a**,**b**) spray drying, (**c**,**d**) electrospray method (**e**,**f**) silicon microfluidic flow focusing device (MFFD). Among these techniques, MFFD produces the most uniform particles (Reprinted from *International Journal of Pharmaceutics*, 467, Kieran Keohane, Des Brennan, Paul Galvin, Brendan T. Griffin, Silicon microfluidic flow focusing devices for the production of size controlled PLGA-based drug loaded microparticles, 60–69, Copyright (2014), with permission from Elsevier [80]).

Emerging emulsification technologies, such as membrane emulsification and microfluidics, have been developed to overcome the limitations encountered by conventional methods. Emulsification that uses membranes with a defined pore size is able to create uniform particles, and this method can consistently reproduce PLGA particles with a low polydispersity compared to the conventional double emulsion method (Figure 7A–D) [78]. In microfluidics, PLGA particles are synthesised in a miniature device to gain better control of the mixing rate, heat, and mass transfer [79]. Progesterone-loaded PLGA microspheres prepared by microfluidics show a narrower size distribution compared to that obtained using the emulsion solvent evaporation technique [27]. To facilitate the reading, a comparison of the PLGA particles produced by microfluidics vs electrospraying and spray drying methods is illustrated in Figure 7a–f [80]. The microfluidic method offers high reproducibility and production rate for large-scale synthesis [81]. It has been reported that up to hundreds of grams of PLGA nanoparticles can be produced per day using a microfluidic device [71]. This production rate is ideal for clinical studies, but may not be sufficient for industrial production [82]. PLGA microparticles prepared using microfluidics display a slower drug release compared to that of the emulsion solvent evaporation and spray drying techniques, respectively. The delay in drug release can probably be attributed to the limited diffusional mass transport through the core of polymer [80].

Despite advances in the drug delivery field, there is still no “one size fits all” manufacturing process. Each of these processes faces different challenges in creating an ideal particulate for efficient drug delivery. The type of encapsulated drug is also an important consideration when it comes to choosing a preferred manufacturing method. Processing of lipophilic drugs is preferably performed using the single emulsion solvent evaporation, nanoprecipitation, and salting out methods [83,84]. The double emulsion, spray drying, and electrospraying methods can be used to encapsulate both lipophilic and hydrophilic drugs [85, 86,87]. The drugs may remain intact or be degraded during the manufacturing process if the processing parameters, such as solvent, temperature, stirring rate, and number of washing and drying steps, are not standardised. The choice of manufacturing process conditions governs the ultimate drug release profiles of the particulates.

Reverse engineering encompasses the elucidation of the post-production composition and important product attributes of commercial PLGA formulations [7,88,89]. The gathered data enable scientists to map the formulation onto the processing parameters, leading to the development of a feasible manufacturing process [88]. In a reverse engineering study of Vivitrol^®^, a significant reduction of PLGA MW (42.25%) was encountered during the preparation of generic formulations [88]. Instead of using the MW of PLGA similar to that of Vivitrol^®^, PLGA of a higher MW was used to produce microspheres in a similar size range. Special attention should be given to pH-sensitive protein or peptide drugs [90], as they are prone to oxidation and acylation by physical stresses during encapsulation. In a reverse engineering study of Bydureon^®^, some insoluble peptide impurities were identified during the processes of encapsulation and in vitro release [89]. The quantity of acylated exenatide by-products was found to increase over time during these processes.

## 7. Strategies to Improve Encapsulation Efficiency and Drug Release

One of the challenges in drug formulations is to encapsulate protein drugs in high loading. The hydrophilic nature of protein drugs makes their partition into the hydrophobic PLGA matrix poor, and they easily leach into the surrounding aqueous phase, resulting in low encapsulation efficiency [91]. Grafting PLGA with other biodegradable polymers, such as polyethylene glycol (PEG), to form a block copolymer with both hydrophilic and hydrophobic sites may be useful for peptide encapsulation [92]. The amphiphilic PLGA–PEG block copolymer can facilitate formation of micelles in the primary emulsion, thus enhances the encapsulation of hydrophilic drugs [93,94].

Hybrid formulations between PLGA and different types of lipids and vegetable oils have been proposed as a new solution for challenging drugs. The hybrid formulations enable higher drug loading, encapsulation efficiency, improving drug bioavailability, and enhancing the overall therapeutic efficacy [95]. The approach was developed by Zhang et al. [96] in designing core-shell hybrid formulation comprising a PLGA core surrounded by lipid-PEG shell. The resulting hybrid formulation has a higher drug encapsulation efficiency in comparison to plain PLGA particles due to the protecting lipid monolayer that helps to maintain the drugs in the PLGA core. The hybrid formulation works particularly well to carry hydrophobic drugs such as paclitaxel, with an encapsulation efficiency beyond 80% [97].

Hydrophobic ion pairing has been adopted in the development of PLGA particulates to deliver charged hydrophilic small molecules and peptides [91]. This method relies on the ion pairing of a charged drug molecule to oppositely charged molecules of a carrier, resulting in the formation of a water-insoluble (hence hydrophobic) uncharged complex. Dextran sulphate has been used to form hydrophobic ion pairing complex with protein drug, resulting in elevated protein entrapment in PLGA nanoparticles of more than 65% [98,99]. Recently, the quality by design approach has been used to evaluate various PLGA particulates for protein therapeutic delivery [10]. This approach enables systemic guiding of the development of PLGA-protein drug products under the relevant authorities.

**Figure 8:**
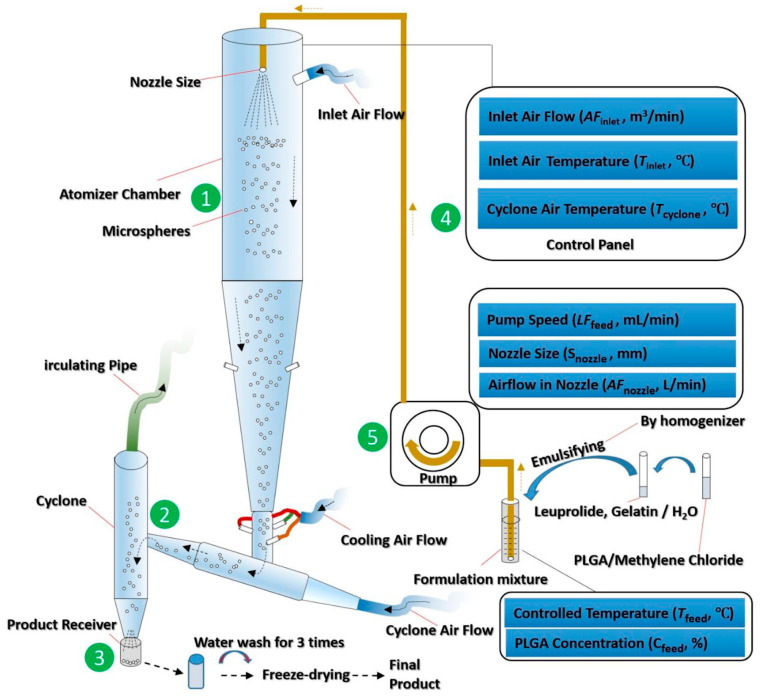
Schematic diagram of a spray drying process for manufacturing drug-loaded PLGA microspheres. Every aspect of the processing parameters, such as heat/mass transfer, inlet air temperature, and drying gas flow rate, requires optimisation in order to achieve the desired final products. (Reprinted from *Journal of Controlled Release*, 321, Nian-Qiu Shi, Jia Zhou, Jennifer Walker, Li Li, Justin K. Y. Hong, Karl F. Olsen, Jie Tang, Rose Ackermann, Yan Wang, Bin Qin, Anna Schwendeman, Steven P. Schwendeman, Microencapsulation of luteinizing hormone-releasing hormone agonist in poly (lactic-*co*-glycolic acid) microspheres by spray-drying, 756–772. Copyright (2020) with permission from Elsevier [87]).

Studies have shown that not only the type and concentration of PLGA, but also the processing parameters, such as solvent type, volume, and excipients, affect the encapsulation efficiency of the resulting drug-loaded PLGA microspheres. Attention should be paid during the production of PLGA therapeutics using a spray drying process. Certain aspects of the processing parameters, such as heat/mass transfer, inlet air temperature, and drying gas flow rate are required to be systematically modified in order to be optimised (Figure 8) [87]. The concentration of PLGA should be adjusted to facilitate water removal during drying [100], while maintaining a certain viscosity [101] to prevent drug leakage from the polymer. Emulsifiers such as polyvinyl alcohol may be used to increase the viscosity of PLGA solution, preventing the outward diffusion of encapsulated drug from the internal PLGA particulates [102]. The addition of stabiliser, such as proline, lysine, or sucrose may further increase the encapsulation efficiency [103].

MFFDs have been reported to outshine the conventional approaches in terms of drug encapsulation performance. The encapsulation efficiency obtained from the processes of MFFDs is significantly higher than that of spray drying and emulsification solvent evaporation methods [80]. Additionally, the amount of drug loss is significantly reduced. Microfluidics and electrospraying methods are preferred over conventional methods as they are able to encapsulate both hydrophobic and hydrophilic drugs [85,104].

The miscibility between PLGA polymer and drug plays an important role in drug loading efficiency of PLGA particulates [105,106,107]. Immiscibility between PLGA and drug can result in phase separation and uneven localisation of the drug within PLGA bulk and on the PLGA surface [108]. The Flory–Huggins interaction parameters have been used to predict the miscibility of various drug-polymer systems [109,110,111]. The model can also be used to predict the miscibility between polymer mixtures [112,113] or between polymer and solvent [114,115]. Molecular modelling is a useful tool for studying the interactions of small-molecule drugs with PLGA of various lactide:glycolide ratios [106]. QronoMetrics^TM^, a computational drug delivery platform that is able to develop LAI formulations may be useful for generic drug companies. However, the modelling depends on sufficient in vitro and in vivo evidence to predict the potential outcomes of a candidate.

High drug loading and low initial release are essential criteria for parenteral controlled drug delivery. Although PLGA has the ability to control drug release up to several months [116,117], but studies have shown that most of the PLGA formulations exhibit initial burst release followed by slow and incomplete release [118]. One of the promising solutions to address these challenges is to integrate PLGA with other polymers, creating block copolymers, composites, and hybrids. Studies on the multiblock PLGA copolymers have shown that water uptake is higher than sole PLGA [119,120]. Additives such as alginate, carboxymethylcellulose, polyvinyl alcohol, poloxomer, gelatin, and chitosan have been embedded in PLGA formulations to extend drug release [121,122].

## 8. Conclusions

Realising the challenges faced in developing generic PLGA-based long-acting drug products, both the pharmaceutical industry and drug regulatory authorities are required to work together closely to overcome these problems. Regular workshops and meetings have been organised by the FDA with industry for the development of complex generic drug products, including those that are PLGA-based. The FDA has also engaged with academics and industry through grants disbursement and contracts offered to support the development of complex generic drug products under the GDUFA programme. Several IVIVCs have been successfully developed in animals for compositionally equivalent PLGA microsphere formulations. Nonetheless, it is still a long way until the establishment of compendial methods with clinical relevance. Furthermore, limited product-specific guidance is available specifically pertaining to bioequivalence studies. To achieve success in PLGA-based long-acting generic drug product development, it is imperative to optimise material, formulation and processing strategies, along with established testing methods in vitro and in vivo.

## Figures and Tables

**Table 1:** PLGA-based long-acting drug products approved by FDA ^†^.

No	Brand Name	API	Indication	Type/ROA	Duration	Manufacturing Method	Year Approved	Ref.
1	Lupron Depot^®^	Leuprolide acetate	Advanced prostate cancer, endometriosis, fibroid	Microsphere; I.M.	1, 3, 4, 6 months	Water-in-oil emulsification	1989, 1995, 1997, 2011	[9,10]
2	Zoladex^®^	Goserelin acetate	Advanced breast cancer in pre- and perimenopausal women, endometriosis and prostate cancer	Solid implant; S.C.	1, 3 months	Hot melt extrusion	1989, 1996	[11]
3	Sandostatin^®^ LAR	Octreotide acetate	Acromegaly	Microsphere; S.C.	1 month	Emulsion solvent evaporation	1998	[9,10]
4	Atridox™	Doxycycline hyclate	Chronic adult periodontitis	In situ gel; Periodontal	1 week	NA	1998	[12]
5	Nutropin Depot^®^	Somatotropin	Growth hormone deficiency	Microsphere; S.C.	1 month	Spray drying	1999	[13]
6	Trelstar^®^	Triptorelin pamoate	Palliative treatment of advanced prostate cancer	Microsphere; I.M.	1, 3, 6 months	Spray drying or Coacervation	2000, 2001, 2010	[10]
7	Arestin^®^	Minocycline HCl	Periodontal disease	Microsphere; Periodontal	2 weeks	NA	2001	[12]
8	Eligard^®^	Leuprolide acetate	Advanced prostate cancer	In situ gel; S.C.	1, 3, 4, 6 months	NA	2002	[12]
9	Risperidal^®^ Consta^®^	Risperidone	Schizophrenia, bipolar I disorder	Microsphere; I.M.	2 weeks	Emulsion solvent evaporation	2003, 2007	[9]
10	Vivitrol^®^	Naltrexone	Alcohol dependence, opioid dependence	Microsphere; I.M.	1 month	Emulsion solvent evaporation	2006	[9]
11	Somatuline^®^ Depot	Lanreotide	Acromegaly, gastroenteropancreatic neuroendocrine tumours, Carcinoid Syndrome	Microsphere; S.C.	1 month	Spray drying	2007	[10]
12	Ozurdex^®^	Dexamethasone	Macular edema	Solid implant; Intravitreal injection	3 months	Spray drying	2009	[11]
13	Propel^®^	Mometasone furoate	Nasal polyps	Solid implant; Sinus implant	1 month	NA	2011	[12]
14	Lupron Depot-PED^®^	Leuprolide acetate	Central precocious puberty	Microsphere; I.M.	1 month	Water-in-oil emulsification	2011	[9]
15	Bydureon^®^	Exenatide	Type 2 diabetes mellitus	Microsphere; S.C.	1 week	Emulsion solvent evaporation or coacervation	2012	[9,10]
16	Lupaneta Pack™	Leuprolide acetate and norethindrone acetate *	Endometriosis	Microsphere; I.M.	1, 3 months	NA	2012	[12]
17	Bydureon^®^ Pen	Exenatide	Type 2 diabetes mellitus	Microsphere; S.C.	1 week	Emulsion solvent evaporation or coacervation	2014	[9,10]
18	Signifor^®^ LAR	Pasireotide	Acromegaly	Microsphere; I.M.	1 month	Emulsion solvent evaporation	2014	[9,10]
19	Bydureon Bcise^®^	Exenatide	Type 2 diabetes mellitus	Microsphere; S.C.	1 week	Emulsion solvent evaporation or coacervation	2017	[9,10]
20	Triptodur™	Triptorelin pamoate	Central precocious puberty	Microsphere; I.M.	6 months	Oil-in-water emulsification/phase separation	2017	[9]
21	Zilretta^®^	Triamcinolone acetonide	Osteoarthritis	Microsphere; Intra-articular	3 months	NA	2017	[12]
22	Sublocade^®^	Buprenorphine	Moderate-to-severe opioid addiction	In situ gel; I.M.	1 month	NA	2017	[12]
23	Sinuva™	Mometasone furoate	Nasal polyps	Solid Sinus implant	3 months	NA	2017	[14]
24	Perseris™	Risperidone	Adult schizophrenia	In situ gel; S.C.	1 month	NA	2018	[12]
25	Fensolvi^®^	Leuprolide acetate	Central precocious puberty	In situ gel; S.C.	6 months	NA	2020	[15]

API: active pharmaceutical ingredient; ROA: route of administration; S.C.: subcutaneous injection; I.M.: intramuscular injection; * norethindrone acetate tablet for oral use; NA: information not available. ^†^ not intended to be fully exhaustive.

**Table 2:** Types of constraints related to the physicochemical properties of PLGA during its formulation and the development of long-acting drug products.

Physicochemical Property	Type of Constraints	Ref.
Lactide:glycolide ratio	Increase hydration rate and drug release rate by increasing proportion of glycolic acid	[39]
Glass transition temperature (Tg)	A higher processing temperature than Tg produces PLGA microspheres with a dense matrix and a smooth surface	[42]
Molecular weight (MW)	Affect drug release kinetics	[43]
Polymer architecture (i.e., star or linear branched chain)	Affect drug release characteristics; difficult to characterise the type of polymer by using conventional gel permeation chromatography and nuclear magnetic resonance spectroscopic methods	[44]
Porosity	High porosity results in faster polymer degradation and drug release	[43]
Particle size	Affect PLGA degradation and drug release	[38]
Drug-polymer interaction	Produce by-products and impurities	[8]

## Data Availability

Not applicable.
